# Nature connectedness and conservation representations among zoo professionals: an exploratory case study

**DOI:** 10.3389/fpsyg.2026.1696216

**Published:** 2026-01-20

**Authors:** Joao Neves, Edgar Ribeiro, Isa Pinho

**Affiliations:** 1 IUCN SSC Center for Species Survival Behavior Change, Albufeira, Portugal; 2 Zoomarine Algarve, Albufeira, Portugal

**Keywords:** conservation representations, environmental attitudes, hybrid organizations, nature connectedness, organizational psychology, organizational sensemaking

## Abstract

Zoological parks with conservation missions must reconcile commercial sustainability with conservation goals within hybrid organizational structures, making employees’ nature connectedness and conservation meaning-making potentially important for achieving their dual mandate. This exploratory single-case study examines links between nature connectedness and conservation representations among employees of a marine-focused zoological park that integrates commercial operations with ex-situ conservation, research, and environmental education. A total of 104 workers completed free word association tasks on “conservation,” a reduced New Environmental Paradigm (NEP) scale, and multidimensional measures of nature connectedness. Reliability analysis supported the use of a refined, 5-item NEP subscale for exploratory purposes. Cluster analysis identified three organizational profiles – Technical Specialists, Cross-functional Workers, and Peripheral Workers – with distinct combinations of conservation expertise, organizational alignment, and nature connectivity. Profiles differed significantly in Personal–Nature Identity and time spent in nature outside work, with Technical Specialists showing unexpectedly lower identity connection and Peripheral Workers reporting the highest nature engagement and technical specificity in their conservation representations. NEP scores, however, did not differ between profiles, and no mediation effects emerged between profiles and environmental attitudes through nature connectedness, aligning with emerging evidence of value–action gaps in complex organizational contexts. These findings suggest that, in hybrid environmental organizations, shared environmental values may coexist with heterogeneous nature connectivity patterns, indicating that organizational challenges may stem less from value conflicts than from structural and representational factors that shape how staff translate conservation commitments into practice.

## 1 Introduction

Modern zoological parks range from primarily commercial attractions to institutions with substantial conservation missions. This study focuses on hybrid environmental organizations that combine commercial operations with *ex situ* conservation, scientific research, and environmental education (Miranda et al., 2023; Keulartz, 2023). While the conservation role of zoos remains contested (Norman and Brando, 2024), organizations with genuine conservation commitments must sustain visitor-based revenue while advancing conservation through research, breeding programs, and public education, making employees’ sensemaking of these dual mandates crucial for mission achievement.

Sustaining these distinct organizational identities while fostering cultures that integrate economic and conservation values requires adaptive strategies and governance mechanisms capable of reconciling potentially conflicting interests. Collective meaning-making thus plays a central role in tension management, shaping how challenges are perceived and how legitimacy is constructed for organizational practices. Shared understanding of commercial and conservation purposes guides sensemaking processes and responses to institutional pressures, facilitating cooperation and practice adaptation when confronting contradictions inherent to the hybrid model. Therefore, analysis of collective meaning-making – that is, social representations – is fundamental to understanding how workers negotiate values, decisions and strategies in pursuing effective integration between commercial and conservation logics. Additionally, the magnitude of psychological connection with nature among these organizations’ workers, involving the degree to which individuals perceive nature as part of themselves, also impacts these organizational processes. The Inclusion of Nature in Self (INS) Theory explains this connection through overlap between concepts of “self” and “nature,” involving cognitive, identity-based and behavioral dimensions that influence sustainable attitudes and actions (Schultz, 2002). However, a gap remains in research regarding how this individual connection interacts with collective social representations within organizations, which shape environmental practices and cultures. This personal connection influences organizational sensemaking by directing more sensitive and integrative interpretations of tensions between commercial and conservation logics, contributing to more legitimate and strategic management in hybrid contexts.

A key challenge in studying nature connectedness among zoo professionals concerns how “nature” is conceptualized. Traditional research often assumes wilderness settings separate from human infrastructure, which may not capture the experiences of staff who work daily with wildlife and design naturalistic habitats (Murphy and Maynard, 2022). This raises questions about how “time in nature” at work compares with recreational nature experiences and how captive wildlife exposure shapes nature connectedness. In this study, “time in nature” is operationalized as recreational activities in natural settings outside work, following established measures (Nisbet et al., 2009; Mayer and Frantz, 2004), while acknowledging that professional interactions with wildlife constitute an additional, unmeasured dimension of nature engagement.

Given the complexity of these psychosocial processes and the limited empirical research in this specific organizational context, this study investigates these relationships within a single paradigmatic case. While this approach limits generalizability, it allows for in-depth investigation of psychological mechanisms that may operate in similar hybrid environmental organizations.

## 2 Literature review

### 2.1 Hybrid environmental organizations: characteristics and institutional tensions

Hybrid environmental organizations such as zoos and aquariums represent a complex organizational phenomenon that combines commercial and environmental/social logics within a single institutional structure (Doherty et al., 2014). These organizations face tensions inherent to their business model, being characterized by the necessity to balance financial objectives with conservation and environmental education purposes (Keulartz, 2023; Miranda et al., 2023). Doherty et al. (2014) identified hybridity as the defining characteristic of social enterprises, manifesting in the simultaneous pursuit of financial sustainability and social purpose. This duality generates specific tensions that extend to organizational mission management, financial resource acquisition and human resource mobilization. In the context of hybrid environmental organizations, such as zoos and aquariums, these tensions manifest particularly in managing conflicting expectations among visitors, stakeholders and conservation objectives (Maynard et al., 2020; Miranda et al., 2023).

### 2.2 The role of workers in organizational meaning construction

Workers in hybrid environmental organizations perform a crucial role in organizational meaning construction, functioning as key agents in articulating between different institutional logics (Weick et al., 2005). Weick et al.’s (2005) organizational sensemaking theory offers a fundamental theoretical lens for understanding how individuals make sense of organizational circumstances and transform them into concrete actions. Sensemaking involves characteristics such as identity, retrospectivity, social activity and plausibility (Weick et al., 2005). These characteristics determine how workers interpret tensions between commercial and social logics, developing adaptive responses that shape organizational culture (Pache and Santos, 2013).

In the context of zoos and aquariums, worker sensemaking is particularly relevant due to the complex nature of the tasks they perform. Professionals in these organizations must steer between commercial expectations (visitor satisfaction, economic viability) and conservation objectives (Carr and Cohen, 2011; Kubarek et al., 2025). This meaning construction process is fundamental to the legitimacy and effectiveness of organizational practices (Whittle et al., 2023). Commercial and conservation logics, while distinct, are not inherently oppositional. Effective conservation increasingly depends on economic sustainability, in line with the three pillars of sustainability (Doherty et al., 2014). Visitor revenue can finance conservation programs, while conservation outcomes enhance legitimacy and visitor appeal. The central challenge for these hybrid organizations is to integrate these logics through governance, culture, and sensemaking processes so that commercial and conservation mandates reinforce rather than undermine each other (Pache and Santos, 2013; Vallaster et al., 2021).

### 2.3 Social representations theory in environmental contexts

Moscovici’s (1961) Social Representations Theory offers an enhanced conceptual framework for understanding how social groups construct shared meanings about environmental issues (Castro, 2006; Moloney et al., 2014; Dézma et al., 2024). Social representations function as practical knowledge systems that guide action and social communication, being particularly relevant for understanding how workers in hybrid environmental organizations interpret conservation challenges.

Social representations operate through two fundamental processes: objectification and anchoring (Moscovici, 1961). Objectification transforms abstract concepts (such as conservation) into concrete images, while anchoring integrates these images into existing knowledge systems (Moscovici, 1961). In organizational contexts, these processes influence how workers perceive and respond to hybrid tensions (Murphy and Maynard, 2022; Vallaster et al., 2021). Application of the social representations theory within environmental contexts has relevance for understanding how different professional groups construct distinct meanings about conservation practices, helping to explain variations in environmental policy adherence and conservation program effectiveness.

### 2.4 Nature connectedness: psychological dimensions and organizational applications

Psychological connection with nature constitutes a multidimensional construct that significantly influences individuals’ environmental attitudes and behaviors (Korach and McConnell, 2021; Barter and Alston-Knox, 2021). These dimensions capture different aspects of the human-nature relationship, from affective and cognitive elements to behavioral ones, with research demonstrating that stronger nature connection typically correlates with increased pro-environmental attitudes and conservation behaviors across diverse populations and contexts (Barragan-Jason et al., 2021).

The relationship between nature connectedness and conservation-oriented behavior in organizations is not linear. Application of INS theory proves particularly important for understanding how individual nature connection influences organizational sensemaking, as professional roles may shape patterns of nature connection across cognitive, identity, and behavioral dimensions of environmental engagement (Barter and Alston-Knox, 2021; Udall et al., 2021; Cuadrado et al., 2023). Personal nature connection directs more sensitive and integrative interpretations of tensions between commercial and environmental logics (Ives et al., 2018), with meta-analytic evidence supporting associations with pro-environmental behavior (Barragan-Jason et al., 2021). However, Kyle and Landon (2023) highlight value–action gaps in complex organizational settings, where even highly nature-connected individuals face constraints from structures, role expectations, and institutional pressures (Vallaster et al., 2021), making these dynamics especially relevant in hybrid environmental organizations.

### 2.5 Theoretical integration, innovation and research questions

Understanding environmental practices and attitudes in hybrid organizations, such as zoos, requires the articulation of two complementary dimensions. The first is the individual connection with nature, which encompasses psychological components—cognitive, experiential, and identity – shaping the subject’s perception and sense of belonging to the natural world (Schultz, 2002; Mayer and Frantz, 2004; Clayton, 2003). This individual connection influences environmental behaviors and attitudes, as well as engagement in conservation actions (Mayer et al., 2009; Nisbet et al., 2009).

The second dimension is that of social representation systems, which are shared collective constructs that shape how groups interpret and attribute meaning to abstract concepts such as “conservation” in institutional contexts (Moscovici, 1961; Jodelet, 2008). In the hybrid organizational environment, these representations guide institutional practices, legitimations and alignments in the face of tensions between commercial and conservationist missions (Vallaster et al., 2021).

These levels do not act in isolation, but rather in a dynamic and complementary path. Individual psychological configurations contribute to forming and transforming social representations, while the latter establish interpretative frameworks that influence the experience and identity of individuals within the organization (Clayton, 2003; Moscovici, 1961). This dialectic is crucial to understanding how environmental professionals negotiate meanings and actions in contexts of strong institutional pressure and complex meaning, such as zoos and aquariums.

The articulation between Social Representation Theory and Organizational Sensemaking provides an integrated perspective for understanding how workers in hybrid organizations such as zoos and aquariums construct meanings about conservation. This integrated theoretical perspective proves particularly relevant for understanding how different professional groups (e.g., specialist technicians, managers, and public-facing staff) develop distinct representations about conservation and how these representations influence sensemaking when facing tensions within the hybrid organizational context.

This theoretical integration suggests three exploratory perspectives for investigation:

1. Potential tensions between technical expertise and personal nature connection.
2. Possible differentiation in conservation representations across organizational roles.
3. Preliminary examination of relationships between individual and collective meaning-making.

Given the exploratory nature and single-case design, these perspectives guide investigation rather than constitute testable hypotheses. Thus, this study addresses three specific research questions: RQ1: Do organizational profiles differ in multidimensional nature connectedness components (INS pictorial, Personal-Nature Identity, Time in Nature Activities)? RQ2: Do organizational profiles exhibit distinct semantic architectures in conservation representations through Categorical Diversity and Technical Specificity of free associations? RQ3: What correlational patterns exist between representational indices, nature connectivity measures, and environmental attitudes within this organizational context?

## 3 Methods

### 3.1 Design and theoretical foundation

The present study adopted a cross-sectional design of quantitative nature, applying a questionnaire survey to the universe of workers at Zoomarine Algarve, a zoo with strong focus on marine life conservation. This methodological approach empirically operationalizes the articulation between Moscovici’s (1961) Social Representations Theory and Weick et al.’s (2005) Organizational Sensemaking theory, allowing the study of how social representations of conservation are structured and differentiated according to specific organizational positions.

### 3.2 Organizational context and data collection procedures

Zoomarine represents a paradigmatic hybrid environmental organization, operating simultaneously as an entertainment theme park and conservation center in the south of Portugal. Established in 1991, the organization combines commercial objectives with conservation and environmental education missions, creating the institutional tensions characteristic of hybrid organizations that make it suitable for this investigation.

Data collection occurred through an online voluntary questionnaire during April 2025, with invitations sent to all workers (*N* = 319) by the Human Resources Department. Two follow-up reminders were conducted at 1.5-week intervals, achieving a 32.6% response rate (*N* = 104). For analysis purposes, organizational directions were classified into technical directions (Conservation & Science, Zoological) directly related to conservation activities, and support directions (Operations, Marketing, Human Resources, Financial) providing organizational support functions.

This single-case study design was chosen for its value in examining complex psychosocial processes within a well-defined organizational context, enabling detailed investigation of mechanisms that may inform theoretical development for future multi-site studies.

### 3.3 Measurement instruments

#### 3.3.1 Free association task

At the beginning of the questionnaire, participants were requested to evoke three words related to the concept “conservation.” This procedure (free association task) aimed to access the semantic structure and thematic content of conservation representations, enabling analysis of categorical diversity and technical specificity across organizational profiles. This study employs basic categorical content analysis of free associations to capture preliminary semantic structures, with the understanding that rigorous social representations research typically requires multi-method approaches and longitudinal designs. Given the exploratory nature of this single-case study, the application of Social Representations Theory is intentionally simplified rather than aiming for a comprehensive representational analysis. The present approach should therefore be understood as a preliminary exploration of representational dimensions rather than a comprehensive social representations study.

Participants produced three evocations to ‘conservation’. Evocations were coded into five categories (technical–scientific; practical–everyday; emotional–evaluative; organizational–contextual; other) using a coding manual. Categorical Diversity (1–4) equals the number of distinct categories across the three evocations; Technical Specificity (0–1) is the proportion of technical–scientific evocations. Detailed coding rules and illustrative examples are provided in Supplementary Table S7.

#### 3.3.2 New environmental paradigm scale (NEP)

We employed a reduced 6-item NEP short form to mitigate survey fatigue and sustain participant motivation within our organizational questionnaire context. This approach is supported by Hawcroft and Milfont’s (2010) meta-analysis of 69 studies across 36 countries, which demonstrates that 6-item versions are frequently used and yield systematically higher scores than the 15-item scale while maintaining acceptable psychometric properties in time-constrained contexts. We, however, acknowledge that scale length and item selection systematically affect scores and dimensional structure. Our reliability analysis revealed a Cronbach’s *α* = 0.57 for the complete 6-item scale, which improved to *α* = 0.63 when excluding the problematic reverse-coded item NEP3 (see Supplementary Table S8) – a finding consistent with literature documenting difficulties with reverse-worded items in the NEP scale (Amburgey and Thoman, 2012). Consequently, we utilized the 5-item pro-environmental subset (excluding NEP3) in our final analyses. We transparently report the exact items used, provide sample-specific psychometric properties, and interpret all NEP findings as exploratory, recognizing they are not directly comparable to studies using the full 15-item instrument.

#### 3.3.3 Inclusion of nature in self scale (INS)

Nature connectedness was assessed using a multidimensional approach combining the original INS pictorial scale (1–7), which measures perceived self-nature identity overlap, with two complementary author-devised measures developed based on Schultz’s (2002) theoretical framework: Personal–Nature Identity (1–5; “To what extent do you feel that your personal identity is connected to nature?”) capturing the identity dimension, and Time in Nature Activities outside work (1–5; “How much time do you spend in natural environments outside of work?”) capturing behavioral engagement. Content validity was established through expert review, with convergent validity evidenced by moderate-to-strong intercorrelations (INS-Personal–Nature Identity, *r* = 0.60; INS-Time in Nature Activities, *r* = 0.41; Personal–Nature Identity-Time in Nature Activities, *r* = 0.32; all *p* ≤ 0.01). These single-item measures, while lacking comprehensive psychometric validation, collectively capture different psychological dimensions of nature connectivity. A Composite INS measure using the standardized mean of all dimensions provided an integrated indicator of multidimensional human-nature connectivity.

#### 3.3.4 Rationale for instrument selection

The multidimensional assessment approach reflects recognition that nature connectedness and environmental attitudes are complex constructs defined in diverse ways (Nisbet et al., 2009; Schultz, 2002). Instrument selection balanced theoretical alignment, psychometric support, and feasibility within organizational constraints. Free word association was used as a low-burden method to access social representations of conservation, complementing quantitative measures by revealing employees’ semantic networks (Moloney et al., 2014). INS-based measures captured cognitive overlap, identity, and behavioral engagement with nature (Schultz, 2002; Nisbet et al., 2009), while a reduced NEP scale indexed ecological worldview (Dunlap et al., 2000). These choices involve researcher discretion, and coding of free associations necessarily reflects interpretive judgments; bias was mitigated through structured coding procedures, acknowledging that complete objectivity in qualitative analysis is unattainable (Jodelet, 2008).

### 3.4 Power analysis and sample size justification

Given the nature of this investigation and the constraints of single-case methodology, sample size was determined based on previous research in organizational environmental psychology. While the obtained sample of *N* = 104 provides adequate power for primary analyses within this organizational context (Baruch and Holtom, 2008), the single-site design limits broader generalizability. Cohen’s (2013) conventions suggest that for MANOVA with 3 groups and 9 dependent variables, a minimum sample of *N* = 84 would be required to detect medium effect sizes (*f* = 0.25) with power = 0.80 and *α* = 0.05. The obtained sample of *N* = 104 exceeds this minimum requirement, providing adequate power for the primary analyses.

### 3.5 Data quality and missing data analysis

The final analytical sample comprised 104 participants with complete data on all primary measures, representing 32.6% of the organizational workforce (*N* = 319) at the time of data collection. Data completeness varied across measures, with free association tasks showing minimal missing data (≤5% per position: 100% for first word, 98.1% for second word, and 95.2% for third word). The NEP 5-item scale and INS measures demonstrated full completeness (100%) across all items due to the mandatory response format once participants initiated the questionnaire. Cases with complete data on key variables were retained for primary analyses, with no sensitivity analyses for missing data imputation required given the complete data availability for all primary measures.

For measures with inadequate statistical power (NEP 5-item: 1-*β* = 0.44; INS pictorial: 1-*β* = 0.52), we report results but refrain from interpreting null findings as evidence of absence.

### 3.6 Analytical procedures

#### 3.6.1 Clustering analysis

To identify distinct organizational profiles, hierarchical clustering was conducted on standardized variables [specialized training, self-perceived conservation knowledge (1–5), participation in conservation activities (binary), organizational alignment (1–5), and entertainment-conservation compatibility (1–5)] using Ward’s linkage method with squared Euclidean distance. The three-cluster solution was selected based on dendrogram elbow inspection, interpretability criteria, and validation procedures including silhouette analysis (mean width = 0.31), bootstrap stability (78.3% recovery rate, 1,000 replications), and comparison with 2–4 cluster alternatives. Cluster distinctiveness was confirmed through discriminant analysis yielding moderate classification accuracy of 50.0% (cross-validated 44.2%; chance = 33.3%), indicating distinguishable though overlapping profiles. Cross-validation using INS dimensions and NEP 5-item showed consistent discriminative patterns, supporting the organizational relevance of the identified profiles.

#### 3.6.2 Categorical content analysis

Free associations underwent systematic categorical content analysis, developing a thematic taxonomy distinguishing five structuring dimensions: technical-scientific (specialized vocabulary, scientific concepts), practical-everyday (concrete actions, observable behaviors), emotional-evaluative (feelings, values, and motivations), organizational-contextual (references to Zoomarine, organizational structures) and others/unclassifiable. For each participant, Categorical Diversity indices and Technical Specificity were calculated, quantitatively operationalizing the semantic structure of conservation social representations.

#### 3.6.3 Differentiated descriptive analyses

An exhaustive characterization of the identified organizational profiles was conducted through the new instrumental dimensions. This characterization included distributions of INS measures (Pictorial, Personal-Nature Identity, Time in Nature Activities), attitudinal profiles associated with NEP 5-item, patterns of thematic categorization of evocations, and sociodemographic and organizational indicators.

#### 3.6.4 Multivariate analysis of variance (MANOVA)

MANOVA was applied to test simultaneous differences between the three organizational profiles in NEP 5-item and in the three complementary INS measures. This approach allowed examination of whether profiles differed significantly in the multivariate set of environmental orientations and identity connections with nature, controlling for intercorrelations between dependent variables. Subsequent univariate ANOVA analyses identified specific contributions of each dimension to differentiation between profiles.

#### 3.6.5 Mediation analyses

Mediation models were tested to examine the mediating role of nature connection (INS) in relationships between organizational profiles and environmental orientations (NEP 5-item). Simple mediation models (Hayes PROCESS Model 4) tested indirect effects of organizational profiles (X) on NEP 5-item (Y) through INS components (M), using bias-corrected bootstrap confidence intervals (5,000 samples) and controlling for education level and organizational tenure. This approach enables understanding of underlying psychosocial mechanisms through which different organizational profiles generate differentiated attitudinal configurations about conservation.

#### 3.6.6 Statistical software

All analyses were conducted using IBM SPSS Statistics version 26.0. Mediation analyses were operationalized through the PROCESS v.4.2 extension (Hayes, 2017).

### 3.7 Ethical considerations

The study was previously approved by the Ethics Committee of the University of Algarve (CEUAlg No. 64/2025). All procedures performed in this study respected the ethical principles of the American Psychological Association (APA) and national regulations in force regarding data protection. Procedural transparency, participant confidentiality, informed consent, and clear separation between researcher role and organizational function were ensured (Blackstone, 2018).

## 4 Results

### 4.1 Sample characterization

The sample included 104 participants with balanced gender distribution, mean age of 37.2 years (±10.4) and age concentration in the 20–40-year ranges (Supplementary Table S1). The educational profile revealed predominance of higher qualifications, with environmental-related training in 17.3% of cases. Professional stratification concentrated mostly in technical and supervisory positions. Data quality assessment confirmed minimal missing data across measures (≤5% for free associations, 0% for standardized scales), with the final analytical sample maintaining representativeness of the organizational workforce.

#### 4.1.1 Identification of organizational profiles

Hierarchical clustering analysis (Ward method) applied to variables of training area, self-perceived conservation knowledge, participation in conservation activities, organizational alignment, and perceived compatibility revealed three distinct organizational profiles capturing the entire sample. Descriptive statistics and distributional properties for all variables by organizational profile are detailed in Supplementary Table S3.

Profile 1 – Technical Specialists (44.2%, *N* = 46) demonstrated elevated specialized training in conservation-related areas, high self-perceived conservation knowledge, and substantial active participation in conservationist activities. This profile showed reduced organizational alignment and concentrated predominantly in technical directions. In nature connectivity patterns, Technical Specialists exhibited significantly lower Personal-Nature Identity (*M =* 3.54) despite their technical expertise. Their conservation representations showed sophisticated temporal evolution from practical dominance (58.7% initial evocations) to emotional development (peak 36.4% in second evocations) while maintaining lower Technical Specificity (*M =* 0.29).

Profile 2 – Cross-functional Workers (45.2%, *N* = 47) characterized by balanced distribution between technical and support directions, presented intermediate training and moderate technical knowledge but demonstrated elevated participation in conservation activities and strong alignment with organizational values. This profile showed higher Personal-Nature Identity (*M =* 4.02) and moderate nature connectivity patterns. Their conservation representations exhibited balanced architectures with stable representational evolution across practical, technical, and emotional dimensions.

Profile 3 – Peripheral Workers (10.6%, *N* = 11) constituted the minority group with reduced specific conservation training and low active participation in conservation activities but paradoxically demonstrated elevated organizational alignment and high perceived compatibility between entertainment and conservation dimensions, mostly within organizational support directions. Despite lower formal training, this profile showed the highest Technical Specificity (*M =* 0.49) and greatest time investment in nature activities outside work (*M =* 3.82).

### 4.2 Nature connection profiles (RQ1): organizational profile differences in multidimensional nature connectedness

RQ1 examined whether organizational profiles differ in nature connectedness components (INS pictorial, Personal-Nature Identity, Time in Nature Activities). MANOVA revealed significant multivariate differences between organizational profiles across nature connectedness dimensions. Personal-Nature Identity demonstrated the largest effect size ( $\eta_{p}^{2}$ =0.096), followed by Technical Specificity ( $\eta_{p}^{2}$ =0.073) and Time in Nature Activities ( $\eta_{p}^{2}$ =0.058). Technical Specialists showed lower scores in Personal–Nature Identity (see Table 1 for complete statistics).

**Table 1:** Multidimensional nature connectedness and representational indices across organizational profiles of zoo workers.

Psychological dimension	Profile 1	Profile 2	Profile 3	*F*	*p*	$\eta_{p}^{2}$
NEP orientation (5-item mean)	4.02 (0.21) [3.62, 4.40]	4.01 (0.20) [3.62, 4.40]	4.01 (0.20) [3.62, 4.40]	2.293	0.106	0.035
INS (pictorial)	4.78 (1.13) [4.45, 5.12]	5.36 (1.36) [4.96, 5.76]	5.36 (1.36) [4.96, 5.76]	2.693	0.073	0.051
Personal identity-nature	3.54 (0.72) [3.33, 3.75]	4.02 (0.79) [3.79, 4.25]	4.00 (0.45) [3.71, 4.29]	5.350	0.006	0.096
Time activities in nature	3.15 (0.87) [2.90, 3.40]	3.45 (0.85) [3.20, 3.70]	3.82 (0.87) [3.25, 4.39]	3.122	0.048	0.058
Categorical diversity	1.17 (0.22) [0.62, 1.71]	1.26 (0.19) [0.79, 1.73]	0.95 (0.27) [0.29, 1.61]	0.331	0.719	0.006
Technical specificity	0.31 (0.30) [0.21, 0.41]	0.37 (0.32) [0.27, 0.47]	0.49 (0.38) [0.24, 0.73]	3.954	0.022	0.073

Values are *M*(SD) [95% CI]. *F*(2,101). $\eta_{p}^{2}$ = partial eta squared. Two-tailed tests. NEP = New Environmental Paradigm (5-item pro-environmental subset); INS (pictorial) = Inclusion of Nature in Self. Assumptions were acceptable (residual normality, homogeneity; Box’s *M* non-significant). Given exploratory nature and multiple comparisons, findings should be interpreted as hypothesis-generating rather than confirmatory.

From the organizational perspective, categorization of nature connectivity variables revealed predominance of high connectivity (70.2% of sample), contrasting with only 5.8% evidencing low connection and 24.0% in neutral position (Supplementary Table S2).

### 4.3 Semantic structures of conservation representations (RQ2): profile differences in representational architectures

Three independent coders classified 94 unique lexical units using five pre-defined categories, achieving moderate reliability (Fleiss’ kappa = 0.579, 95% CI: 0.513–0.646). Overall distribution showed predominance of practical-everyday content (53.8% first evocation), followed by technical-scientific (34.6%) and emotional-evaluative content (8.7%) (see Supplementary Table S4).

RQ2 examined whether organizational profiles exhibit distinct semantic architectures through Categorical Diversity and Technical Specificity of free associations. Profiles exhibit differentiated semantic architectures in conservation representations, with significant differences in Technical Specificity (*p =* 0.022, $\eta_{p}^{2}$ =0.073) but no significant differences in Categorical Diversity (*p =* 0.719) (Table 1).

Technical Specialists showed the lowest Technical Specificity (*M =* 0.31) and demonstrated sophisticated temporal processing: practical-everyday dominance (58.7%) shifting to emotional-evaluative prevalence (36.4% second evocation) then technical-scientific (33.3% third evocation) (Table 2).

**Table 2:** Distribution of conservation free-association categories by organizational profile and evocative position.

Semantic category	Profile 1	Profile 2	Profile 3	Total
First evocation				
Technical-scientific	14 (30.4%)	18 (38.3%)	4 (36.4%)	36 (34.6%)
Practical-everyday	27 (58.7%)	22 (46.8%)	7 (63.6%)	56 (53.8%)
Emotional-evaluative	3 (6.5%)	6 (12.8%)	0 (0.0%)	9 (8.7%)
Organizational-contextual	2 (4.3%)	0 (0.0%)	0 (0.0%)	2 (1.9%)
Others/unclassifiable	0 (0.0%)	1 (2.1%)	0 (0.0%)	1 (1.0%)
Second evocation				
Technical-scientific	15 (34.1%)	18 (38.3%)	7 (63.6%)	40 (39.2%)
Practical-everyday	13 (29.5%)	17 (36.2%)	1 (9.1%)	31 (30.4%)
Emotional-evaluative	16 (36.4%)	9 (19.1%)	1 (9.1%)	26 (25.5%)
Organizational-contextual	0 (0.0%)	2 (4.3%)	2 (18.2%)	4 (3.9%)
Others/unclassifiable	0 (0.0%)	1 (2.1%)	0 (0.0%)	1 (1.0%)
Third evocation				
Technical-scientific	14 (33.3%)	16 (34.0%)	5 (50.0%)	35 (35.4%)
Practical-everyday	13 (31.0%)	14 (29.8%)	0 (0.0%)	27 (27.3%)
Emotional-evaluative	6 (14.3%)	8 (17.0%)	3 (30.0%)	17 (17.2%)
Organizational-contextual	8 (19.0%)	2 (4.3%)	1 (10.0%)	11 (11.1%)
Others/unclassifiable	1 (2.4%)	7 (14.9%)	1 (10.0%)	9 (9.1%)

Absolute and relative frequencies of technical-scientific, practical-everyday, emotional-evaluative, organizational-contextual, and other/unclassifiable evocations for first, second, and third words across the three profiles.

Cross-functional Workers exhibited balanced architecture with moderate Technical Specificity (*M =* 0.37), maintaining consistent technical and practical content across evocations with gradual emotional increases (Table 2).

Peripheral Workers showed highest Technical Specificity (*M =* 0.49) across evocations exhibiting dramatic transformation from practical dominance (63.6% → 0.0%) to substantial technical development (36.4% → 63.6% → 50.0%) and progressive emotional emergence (0.0% → 9.1% → 30.0%) (Table 2).

### 4.4 Relational analyses (RQ3): associations between representational indices, nature connectivity, and organizational variables

RQ3 examined correlational patterns between representational indices, nature connectivity measures, and environmental attitudes. Correlational analysis revealed theoretically congruent associations between representational profiles, psychological dimensions and organizational characteristics (Table 3). Key associations emerged (*p <* 0.01): Personal-Nature Identity ↔ Representational Profile (*r =* 0.27), Educational Level ↔ Representational Profile (*r =* 0.27), Personal-Nature Identity ↔ INS pictorial (*r =* 0.60), INS pictorial ↔ Time Nature Activities (*r =* 0.41), and Personal-Nature Identity ↔ Time Nature Activities (*r =* 0.32). Additional correlations showed minor significance (*p <* 0.05): NEP Orientation ↔ Representational Profile (*r* = 0.20), Time Nature Activities ↔ Representational Profile (*r =* 0.24), NEP Orientation ↔ Personal-Nature Identity (*r* = 0.22), Education Level ↔ Technical Specificity (*r =* 0.23) and Education Level ↔ Organizational Tenure (*r* = −0.24).

**Table 3:** Pearson correlations between nature connectedness, conservation representations, environmental attitudes, and organizational characteristics among zoo workers.

Variable	1	2	3	4	5	6	7	8
1. Representational profile	–							
2. NEP orientation (5-item mean)	0.20^*^	–						
3. INS (pictorial)	0.18	0.12	–					
4. Personal-nature identity	0.27^**^	0.22^*^	0.60^**^	–				
5. Time nature activities	0.24^*^	0.01	0.41^**^	0.32^**^	–			
6. Categorical diversity	−0.10	−0.16	0.01	−0.11	0.05	–		
7. Technical specificity	0.16	0.10	0.03	0.04	−0.01	−0.19	–	
8. Educational level	0.27^**^	−0.01	−0.07	0.05	0.01	0.10	0.23^*^	–
9. Organizational tenure	−0.05	0.01	0.10	0.00	0.04	0.04	−0.05	−0.24^*^

*N* = 104. ^*^p < 0.05, ^**^p < 0.01. Given the exploratory nature of this investigation, uncorrected p-values are reported alongside effect size indicators to balance discovery potential with interpretive rigor.

#### 4.4.1 Interpretation of associative patterns

The observed correlations should be interpreted strictly as associations within this organizational context. The cross-sectional design precludes any causal inferences. Markedly, the modest effect sizes (*r* ranging from 0.20 to 0.32 for significant correlations) indicate that these relationships, while statistically detectable, explain limited variance in outcomes (4–10% of shared variance). Given the exploratory nature of this investigation and multiple comparisons performed, these correlational patterns should be considered hypothesis-generating rather than confirmatory.

The absence of significant indirect pathways in our exploratory models (see Supplementary Figure S1 for attempted configurations) suggests that relationships between organizational profiles and environmental orientations operate through complex, non-linear configurations rather than simple mediational chains. Future longitudinal research is needed to disentangle these relationships.

### 4.5 Environmental attitudes: exploratory analysis

Environmental attitudes were examined as an outcome variable using the NEP 5-item scale. Environmental orientations showed no significant differences between profiles [*F*(2,101) = 2.293, *p =* 0.106, $\eta_{p}^{2}$ =0.035]. All profiles demonstrated consistently ecocentric positions (means > 3.8), suggesting shared environmental values across organizational roles despite differences in nature connectivity patterns. Cross-functional Workers showed the highest environmental orientation (*M* = 4.02), followed by Peripheral Workers (*M* = 3.98) and Technical Specialists (*M* = 3.83).

#### 4.5.1 Associations with nature connectivity

Regression analysis using the 5-item NEP score indicated that the three predictors together explained 7.0% of the variance in environmental attitudes [*R^2^* = 0.07, *F*(3,92) = 2.30, *p* = 0.08]. None of the individual predictors reached statistical significance levels. Sensitivity analyses comparing 6-item and 5-item NEP configurations showed substantively identical patterns (see Supplementary Tables S5, S6).

#### 4.5.2 Statistical power and effect size analysis

Post-hoc power analyses were conducted using G*Power 3.1.9.7 (Faul et al., 2007) to evaluate the adequacy of the obtained sample size (*N* = 104). The MANOVA achieved a good power (1-*β* = 0.87–0.90) for detecting multivariate differences between profiles (*η^2^* = 0.08–0.14). Individual ANOVAs revealed considerable variations in statistical power among the variables analyzed. The test showed adequate power for: INS Personal-Nature Identity (1-*β* = 0.82, *η^2^* = 0.095, *p* = 0.007) and INS Time on Nature Activities (1-*β* = 0.59, *η*^2^ = 0.059, *p* = 0.047).

However, the analyses showed insufficient statistical power for: NEP 5-item (1-*β* = 0.44, *η*^2^ = 0.042, *p* = 0.118) and INS (pictorial) (1-*β* = 0.52, *η*^2^ = 0.075, *p* = 0.073).

These power limitations suggest that non-significant or marginally significant results for the NEP 5-item and INS (pictorial) variables should be interpreted with caution, as they may reflect insufficient statistical power rather than true null effects. For future investigations, it is recommended to increase the sample size to improve the ability to detect small to medium magnitude effects.

## 5 Discussion

This exploratory case study identified preliminary patterns in nature connectedness and conservation representations within a single hybrid organization. While findings are strictly limited to this organizational context and cannot establish causality, three tentative observations emerged that align with existing theoretical frameworks while requiring validation through more robust research designs.

### 5.1 Observed organizational patterns and nature connectivity

The identification of three distinct organizational profiles with differentiated nature connectivity patterns provides initial evidence for complex relationships between professional roles and environmental engagement. Technical Specialists demonstrated lower Personal-Nature Identity (*M* = 3.54) despite presumed expertise, while Peripheral Workers showed elevated connectivity (*M* = 4.00) alongside unexpectedly high Technical Specificity (*M* = 0.49) in conservation representations.

This counter-intuitive pattern resonates with emerging research on professional identity management in demanding organizational contexts. Anisman-Razin et al. (2025) document how specialized professionals may employ “doing distance” strategies—intentional psychological distancing that preserves objectivity and professional authority when facing complex challenges. Similarly, Li and Chen (2019) identify paradoxical relationships between expertise and psychological distance, suggesting that technical knowledge can increase critical awareness of organizational limitations, potentially leading to protective cognitive strategies.

The multidimensional dissociation observed between cognitive, experiential, and identity components of nature connection aligns with Schultz’s (2002) theoretical framework and recent empirical work by Cuadrado et al. (2023) demonstrating that different occupational groups exhibit varying relationships between environmental engagement dimensions. Barter and Alston-Knox (2021) similarly found that proximity and control mechanisms influence how individuals translate environmental values into organizational outcomes.

These preliminary observations suggest that organizational roles may be associated with distinct psychological configurations of environmental engagement rather than linear progressions based on expertise or hierarchical position. However, the modest effect sizes ( $\eta_{p}^{2}$ =0.058–0.096) indicate these relationships explain limited variance, necessitating replication with larger samples and validated measures.

### 5.2 Representational architectures and professional contexts

The observed differentiation in conservation representations across organizational profiles provides initial support for Social Representations Theory applications in hybrid organizational contexts (Moscovici, 1961; Castro, 2006). Technical Specialists’ lower Technical Specificity (*M* = 0.31) combined with sophisticated temporal processing – evolving from practical emphasis (58.7%) to emotional integration (36.4%) to technical articulation (33.3%) – suggests complex representational strategies that transcend simple expertise-specificity relationships.

This pattern finds theoretical support in Cognitive Load Theory frameworks (Patel and Alismail, 2024), which propose that complex organizational demands may be managed through differentiated processing strategies. Brando et al. (2023) identify “empathic strain” among conservation professionals, potentially explaining how technical specialists develop adaptive representational approaches that balance emotional engagement with professional sustainability.

The positive correlation between educational level and Technical Specificity (*r* = 0.23, *p* < 0.05), while modest, aligns with Kubarek et al.’s (2025) observations about technical specialization dynamics in zoo contexts. However, this relationship explains only 5% of representational variance, indicating that formal training is merely one factor influencing conservation articulation. The negative correlation between education and organizational tenure (*r* = −0.24, *p* < 0.05) may reflect generational shifts in professional preparation, consistent with broader trends in environmental education (Kyle and Landon, 2023).

Peripheral Workers’ achievement of highest Technical Specificity despite lower formal training resonates with Granovetter’s (1973) social network theory, suggesting that different structural positions may exploit distinct informational advantages. This pattern also aligns with Udall et al.’s (2021) meta-analysis demonstrating that environmental identity can manifest through diverse pathways beyond formal training.

### 5.3 Environmental attitudes and organizational dynamics

Environmental attitudes showed no significant differences between profiles [*F*(2,101) = 2.293, *p* = 0.106], though statistical power was inadequate (1-*β* = 0.44) for confident interpretation of this null finding. All profiles demonstrated consistently pro-environmental orientations (means >3.8), suggesting shared environmental values across organizational roles despite differences in nature connectivity patterns. Mediation analyses confirmed no indirect effects of profile membership on NEP scores through nature connectedness. This pattern, shared environmental values amid differing personal connections and representational architectures (e.g., lower technical specificity in Technical Specialists vs. higher in Peripheral Workers), aligns with value–action gap accounts (Kyle and Landon, 2023), suggesting challenges in hybrid organizations stem more from structural barriers than value conflicts.

This uniform environmental commitment echoes Murphy and Maynard’s (2022) work on workplace attachment among zoo professionals and Miranda et al.’s (2023) observations on conservation-oriented organizational cultures in hybrid settings. The absence of attitudinal differences despite connectivity variations supports Ives et al.’s (2018) view that environmental engagement can follow multiple pathways while resting on shared underlying values. The lack of mediation effects between organizational profiles and environmental attitudes via nature connectivity suggests more complex, non-linear configurations than simple indirect paths, in line with Vallaster et al.’s (2021) account of interacting psychological mechanisms underpinning dynamic capabilities in hybrid organizations.

### 5.4 Theoretical integration and organizational sensemaking

The observed patterns provide preliminary support for integrating Social Representations Theory (Moscovici, 1961) with Organizational Sensemaking frameworks (Weick et al., 2005) in hybrid environmental contexts. The differentiated representational architectures across profiles suggest that different organizational positions may contribute complementary cognitive resources for managing institutional tensions characteristic of hybrid organizations (Doherty et al., 2014; Pache and Santos, 2013).

Technical Specialists’ broader representational approach may facilitate integration across diverse conservation domains, while Cross-functional Workers’ balanced profiles enable bridging between specialized content and organizational communication needs. Peripheral Workers’ precise technical articulation combined with elevated nature connectivity could provide valuable boundary-spanning capabilities, consistent with Maynard et al.’s (2020) research on collaborative networks in zoo organizations. Although numerically small, Peripheral Workers also report the greatest time in nature outside work despite low formal conservation training and conservation activity at the zoo. This suggests an underused pool of conservation-relevant engagement among support-function staff, underscoring the importance of creating structures that allow peripheral workers to participate more directly in conservation initiatives and organizational sensemaking.

This functional complementarity aligns with Whittle et al.’s (2023) framework on language in organizational sensemaking and Keulartz’s (2023) analysis of adaptive strategies in contemporary zoos. However, the cross-sectional design prevents determination of whether these represent stable organizational configurations or dynamic responses to institutional pressures.

### 5.5 Practical implications and organizational applications

While these findings require validation in diverse organizational contexts, they offer preliminary insights for human resource management in hybrid environmental organizations. The identification of complementary representational strengths suggests potential value in recognizing diverse cognitive contributions rather than expecting uniform environmental engagement patterns.

Professional development programs might benefit from differentiated approaches that leverage distinct connectivity configurations. Technical Specialists may benefit from autonomy that respects protective psychological strategies, while Peripheral Workers might be engaged through experiential conservation activities that capitalize on their authentic environmental connection (Carr and Cohen, 2011; Norman and Brando, 2024).

Performance evaluation systems might recognize different forms of conservation contribution rather than applying uniform criteria that could penalize legitimate adaptive strategies. This aligns with recent research on agency and wellbeing in zoo contexts (Norman and Brando, 2024) and broader frameworks for managing institutional tensions in hybrid organizations.

### 5.6 Study limitations and interpretive constraints

Several critical limitations significantly constrain interpretation of these findings. The single-organizational case study design limits generalizability beyond this specific context. Statistical power was inadequate for several key variables (NEP 5-item: 1-*β* = 0.44; INS pictorial: 1-*β* = 0.52), meaning non-significant results may reflect Type II errors rather than true null effects.

The modest reliability of environmental attitude measures (NEP 5-item *α* = 0.63) and lack of validation for author-devised nature connectivity measures introduce measurement uncertainty. Cross-sectional design prevents causal inferences about identified relationships, while voluntary participation (32.6% response rate) may have introduced self-selection bias toward environmentally engaged workers.

Effect sizes, while statistically detectable, explain limited variance in outcomes, indicating that organizational profiles account for small portions of individual differences in nature connectivity and representational patterns. Cultural and regulatory contexts specific to the Zoomarine setting may limit transferability to other regions or organizational types.

### 5.7 Future research directions and theoretical development

The exploratory nature of this investigation makes replication and extension the highest priorities for future research. Multi-site studies across diverse hybrid environmental organizations are essential to establish generalizability of identified organizational profiles and psychological mechanisms. Cross-cultural validation is particularly important given potential variations in nature connectivity and organizational sensemaking processes.

Longitudinal investigations could examine temporal stability of psychological configurations and track how organizational changes affect representational processes. Mixed-method approaches combining quantitative frameworks with ethnographic observation would provide richer understanding of underlying mechanisms suggested by these preliminary patterns.

Validation of the nature connectivity measures developed here, particularly the single-item Personal-Nature Identity and Time in Nature Activities scales, represents a methodological priority. Development of organizational-specific environmental attitude measures might address the reliability concerns identified with the NEP 5-item scale in this context.

## 6 Conclusion

This exploratory case study provides preliminary evidence for differentiated psychological configurations within a hybrid environmental organization, with implications for understanding how professional roles relate to environmental engagement. The identification of complementary representational approaches suggests potential value in recognizing diverse forms of conservation contribution rather than expecting uniform patterns.

However, methodological constraints necessitate cautious interpretation pending replication with validated measures, adequate statistical power, and longitudinal designs across multiple organizational contexts. The modest effect sizes observed indicate that organizational profiles, while statistically detectable, explain limited variance in environmental outcomes.

Future research should prioritize multi-site validation, methodological refinement, and theoretical development to determine whether these preliminary patterns reflect stable organizational phenomena or context-specific artifacts. The integration of Social Representations Theory with Organizational Sensemaking frameworks shows promise for understanding hybrid environmental organizations but requires more robust empirical foundations.

## Data Availability

The raw data supporting the conclusions of this article will be made available by the authors, upon reasonable request.
