# Supplementary material for: Nature connectedness and conservation representations among zoo professionals: an exploratory case study
Source: Front Psychol. 2026 Jan 20;17:1696216. doi: 10.3389/fpsyg.2026.1696216 (PMC12864103; doi:10.3389/fpsyg.2026.1696216)
Supplement: Supplementary file 1 [file Supplementary_file_1.pdf]

Supplementary Material

Table S1 - Sociodemographic and organizational characteristics of zoo employees participating in the study (N = 104).

	**N**	**%**
**Gender Distribution**		
Female	56	53.8
Male	48	46.2
**Age Range**		
20-30 years-old	32	30.8
31-40 years-old	35	33.7
41-50 years-old	25	24.0
51+ years-old	12	11.5
**Educational Qualifications**		
Basic & Secondary Education	36	34.6
Bachelor's & Professional	50	48.1
Master's / Doctorate	18	17.3
**Training Area**		
Environmental-related	18	17.3
Non-Environmental-related	24	23.1
Not specified	62	59.6
**Professional Stratification**		
Assistant	12	11.5
Technical & Supervisor	67	64.4
Management	17	16.3

Direction	8	7.7
**Organisational Tenure**		
≤ 5 years	39	37.5
6–15 years	37	35.6
> 15 years	28	26.9
**Contract Type**		
Seasonal	28	26.9
Permanent	76	73.1
**Departmental Affiliation**		
Support Departments	47	45.2
Technical Departments	57	54.8

Note. Absolute and relative frequencies for gender, age, education, training area, professional category, organizational tenure, contract type, and departmental affiliation.

Table S2. Frequency distributions of categorized nature connectedness and contractual status among zoo employees (N = 104).

**Variable**	**Category**	**N**	**%**
Categorized INS (pictorial)			
	Low (1-3)	6	5.8%
	Neutral (4)	25	24.0%
	High (5-7)	73	70.2%
Strong Environmental Orientation (NEP>3.5 + INS>5)			
	Conventional	34	32.7%
	Strong	70	67.3%
Contractual Modality			
	Seasonal	28	26.9%
	Permanent	76	73.1%

Note. INS pictorial scores categorized into low (1–3), neutral (4), and high (5–7) nature connectedness; "Strong environmental orientation" defined by combined NEP (>3.5) and INS (>5); contractual modality distinguishes seasonal and permanent contracts. Distributional configuration: N=104. High connectivity with nature (70.2%) and strong environmental orientation (67.3%) characterise a globally ecocentric organisational profile.

Table S3. Sociodemographic differentiation of zoo employees by representational profile (Technical Specialists, Cross-functional Workers, Peripheral Workers).

	**Profile 1**	**Profile 2**	**Profile 3**
Gender Distribution			
Female	24 (52.2%)	26 (55.3%)	6 (54.5%)
Male	22 (47.8%)	21 (44.7%)	5 (45.5%)
Educational Qualifications			
Basic & Secondary	23 (50.0%)	10 (21.3%)	3 (27.3%)
Bachelor's & Professional	21 (45.7%)	22 (46.8%)	7 (63.6%)
Master's / Doctorate	2 (4.3%)	15 (31.9%)	1 (9.1%)
Organisational Tenure	2.0 (0.9)	1.8 (0.8)	2.0 (0.4)

Note: Categorical values expressed as N (%). Continuous values as $M(SD)$. Educational Qualifications differed by profile, $\chi^2(4)=17.046$, $p<0.001$.

Table S4. Mean indices of representational complexity and semantic categories of "conservation" by organizational profile.

**Categorical Dimension**	**Profile 1**	**Profile 2**	**Profile 3**	**Total**
Representational Complexity Indices				
Categorical Diversity (1-4)	1.93 (0.65)	1.91 (0.63)	2.00 (0.67)	1.93 (0.64)
Technical Specificity (0-1)	0.31 (0.30)	0.37 (0.32)	0.48 (0.38)	0.36 (0.32)
Semantic Categories (proportions)				
Technical-Scientific	0.67 (0.48)	0.74 (0.44)	0.82 (0.40)	0.71 (0.46)
Practical-Everyday	0.85 (0.36)	0.77 (0.43)	0.64 (0.50)	0.79 (0.41)
Emotional-Evaluative	0.67 (0.48)	0.57 (0.50)	0.55 (0.52)	0.62 (0.49)
Organisational-Contextual	0.24 (0.43)	0.15 (0.36)	0.36 (0.50)	0.21 (0.41)

Representational configuration: Values expressed as *M*(*SD*). N = 104. Differentiated gradation in the dimensions of semantic complexity is consistent with systematic intra-organisational representational heterogeneity.

Table S5 – Extended regression models predicting 6-item NEP scores from nature connectedness and representational indices.

Model	Outcome	Predictor	*B*	SE *B*	*β*	95% CI lower (*B*)	95% CI upper (*B*)	*t*	*p*	VIF	R^2	Adj R^2	*F*(df)	*F p*
A	NEP 6-item	Intercept	4.296	0.048		4.200	4.392	89.50	<0.001		0.074	0.036	13.15 (1,102)	<0.001
A	NEP 6-item	INS (pictorial)	0.28	0.08	0.34	0.12	0.44	3.63	<0.001	1.00				
B	NEP 6-item	Intercept	3.77	0.27		3.23	4.31	13.90	<0.001		0.07	0.04	3.61 (2,101)	0.031
B	NEP 6-item	INS (pictorial)	0.04	0.04	0.09	-0.04	0.11	0.90	0.371	1.16				
B	NEP 6-item	Personal–Nature Identity	0.11	0.06	0.17	-0.02	0.23	1.75	0.083	1.29				

B	NEP 6-item	Time in Nature Activities	-0.02	0.06	-0.03	-0.13	0.09	-0.35	0.730	1.18				
C	NEP 6-item	Intercept	3.769	0.271		3.231	4.307	13.90	<0.001		0.074	0.036	1.97 (4,99)	0.105
C	NEP 6-item	Composite INS	0.01	0.05	0.01	-0.09	0.11	0.13	0.898	1.30				
C	NEP 6-item	Categorical Diversity	-0.05	0.06	-0.07	-0.17	0.07	-0.85	0.397	1.20				
C	NEP 6-item	Technical Specificity	0.01	0.09	0	-0.17	0.19	0.11	0.915	1.20				

Note: Model A = simple regression with pictorial INS only; Model B = multiple regression with separate INS components; Model C = primary model with composite INS and representational indices. β = standardized beta coefficient. NEP = New Environmental Paradigm 6-item mean. Composite INS = standardized mean of pictorial INS, Personal-Nature Identity, and Time in Nature Activities. VIF <2 indicates acceptable multicollinearity levels.

Table S6 – Sensitivity analysis: regression models predicting 5-item pro-environmental NEP scores (excluding NEP3).

Model	Outcome	Predictor	*B*	SE *B*	*β*	95% CI lower (*B*)	95% CI upper (*B*)	*t*	*p*	VIF	R^2	Adj R^2	*F*(df)	*F p*
A	NEP 5-item	Intercept	4.296	0.049		4.201	4.392	87.67	<0.001		0.122	0.114	14.15 (1,102)	<0.001
A	NEP 5-item	INS (pictorial)	0.27	0.08	0.33	0.11	0.43	3.76	<0.001	1.00				
B	NEP 5-item	Intercept	4.182	0.047		4.088	4.277	88.98	<0.001		0.205	0.181	6.73 (3,100)	0.0004
B	NEP 5-item	INS (pictorial)	0.11	0.09	0.14	-0.07	0.29	1.23	0.222	1.16				
B	NEP 5-item	Personal–Nature Identity	0.18	0.10	0.21	-0.02	0.38	1.86	0.066	1.29				

B	NEP 5-item	Time in Nature Activities	0.06	0.08	0.07	-0.10	0.22	0.76	0.448	1.18				
C	NEP 5-item	Intercept	4.296	0.049		4.201	4.392	87.67	<0.001		0.205	0.181	6.43 (3,100)	<0.001
C	NEP 5-item	Composite INS	0.33	0.08	0.33	0.17	0.49	4.16	<0.001	1.30				
C	NEP 5-item	Categorical Diversity	-0.20	0.06	-0.23	-0.31	-0.08	-3.41	0.001	1.20				
C	NEP 5-item	Technical Specificity	-0.18	0.09	-0.16	-0.35	-0.01	-2.03	0.045	1.20				

Note: Replication of regression models using 5-item pro-environmental NEP score (excluding reverse-scored NEP3). Model specifications identical to Table S5. The 5-item configuration showed improved reliability (α=0.63 vs. α=0.57 for 6-item version). Consistent pattern of predictors across both NEP versions supports robustness of findings.

Table S7 – Coding manual for conservation free associations. The table lists operational definitions, decision rules, examples of borderline cases used to compute Categorical Diversity (1–4) and Technical Specificity (0–1).

Category	Operational definition	Decision rules (borderline)
Technical–Scientific	Scientific/technical content: species, diagnoses, methods, habitats, protocols.	Prioritize content over valence; if mixed with emotion, choose technical if specific method/species is present.
Practical–Everyday	Lay actions/routines without jargon; quotidian practices.	Action-oriented but non-technical stays here; if overlaps with policy, prefer practical when micro/lay level.
Emotional–Evaluative	Affect/values/moral judgments without technical detail.	If emotion + generic content, prioritize emotional (e.g., respect for animals).
Organizational–Contextual	Policies, departments, programs, regulations, stakeholder/market context.	If program/department named, choose organizational; if mixed with technique, pick organizational when the unit is the organization.
Other/Unclassifiable	Too vague/idiosyncratic/not fitting criteria even after discussion.	Use sparingly; after coder discussion.

Table S8 - Reliability Analysis of the New Environmental Paradigm (NEP) Scale: Comparative Statistics for 6-item and 5-item Configurations

Scale	*α*	95% CI	Std *α*	*M*	*SD*
NEP 6-item	0.569	[0.435, 0.703]	0.603	25.37	2.72
NEP 5-item	0.629	[0.504, 0.754]	0.642	21.48	2.45

Item-by-Item Analysis (*α* if item deleted)

Item	6 items	5 items	*r* item-total
NEP1: Serious environmental abuse	0.467	-	0.442
NEP2: Delicate balance of nature	0.434	-	0.529
NEP3: Human right to modify environment	0.629	*removed*	0.103
NEP4: Approaching population limit	0.530	-	0.317
NEP5: Equal rights plants/animals	0.527	-	0.327
NEP6: Humans subject to natural laws	0.541	-	0.268

Critical Inter-item Correlations

Item pairs	r	Assessmen t
NEP3 ↔ NEP1	0.021	Very weak
NEP3 ↔ NEP4	- 0.036	Negative
NEP1 ↔ NEP4	0.391	Moderate

NEP1 ↔ NEP2	0.356	Moderate

Note: Cronbach's α with 95% confidence intervals. The 6-item scale includes one reverse-scored item (NEP3) which showed problematic psychometric properties. The 5-item pro-environmental subset excludes NEP3 and demonstrates improved internal consistency.

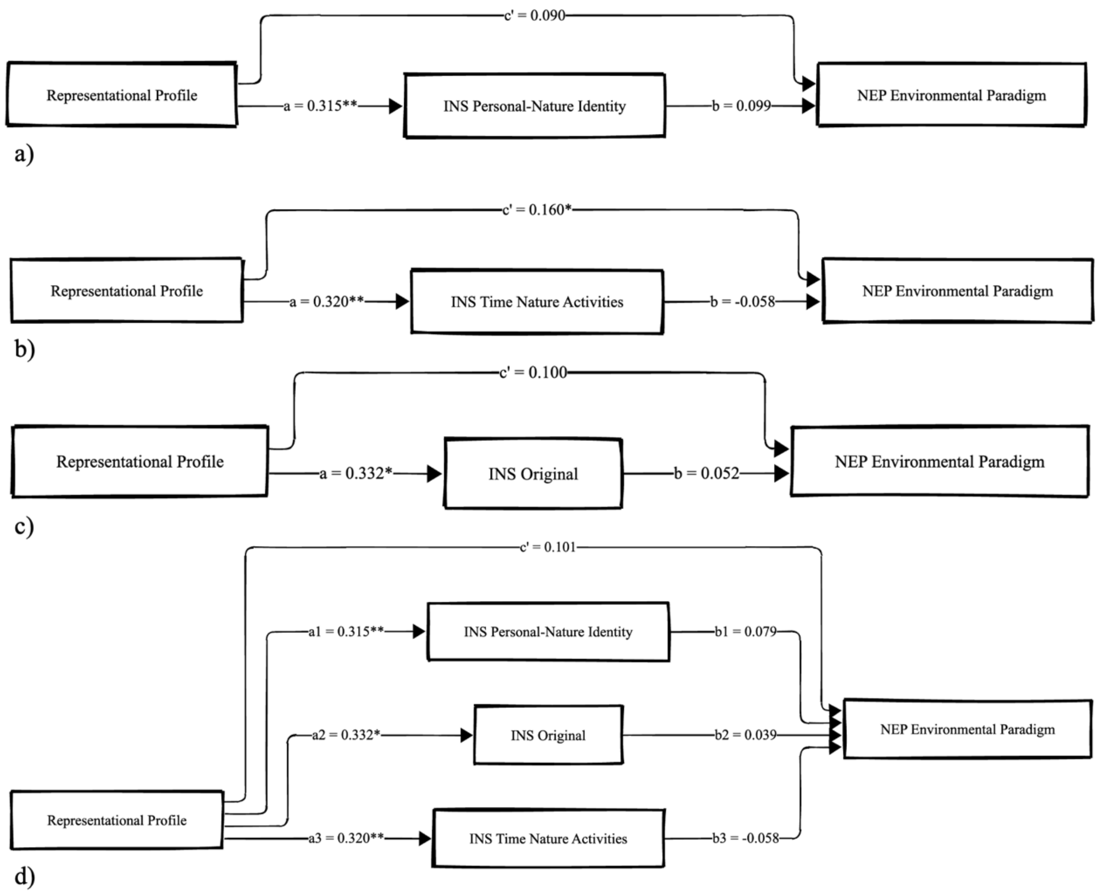

Figure S1. Exploratory Association Models: Organizational Profiles, Nature Connection, and Environmental Paradigms.

Note: Standardized path coefficients are presented. a = effect of independent variable on mediator; b = effect of mediator on dependent variable; c' = direct effect of independent variable on dependent variable, controlling for mediator(s). * p<0.05, ** p<0.01. INS = Inclusion of Nature in Self; NEP = New Environmental Paradigm

Research Questionnaire

Section A: Sociodemographic Characteristics

Age: ___________

Gender:

- [ ] Male
- [ ] Female
- [ ] Other

Educational Level:

- [ ] Basic Education
- [ ] Secondary Education or equivalent
- [ ] Technological Specialization Course
- [ ] Bachelor's Degree *(Which?* ________________)
- [ ] Master's Degree *(Which?* ________________)
- [ ] Doctorate *(Which?* ________________)

Years working at [blinded for review]: ___________

Type of contract:

- [ ] Seasonal
- [ ] Permanent

Current department: ____________________________

Professional category:

- [ ] Director
- [ ] Department Head
- [ ] Section Head
- [ ] Supervisor
- [ ] Technician
- [ ] Assistant

Section B: Conservation

What are the first three words that come to mind when you think of "conservation"? *(Please indicate the words in the order they come to mind)*

1. ______________________________
2. ______________________________
3. ______________________________

To what extent do you have knowledge about conservation?

- [ ] No knowledge
- [ ] Limited knowledge
- [ ] Moderate knowledge
- [ ] Good knowledge
- [ ] Extensive knowledge

Do you participate in nature conservation activities?

- [ ] Yes *(Which ones? ________________)*
- [ ] No

Section C: Environmental Worldviews (NEP Scale)

The following statements relate to general views about the relationship between humans and the natural environment. Please indicate your level of agreement with each of the following statements.

Scale: 1 = Strongly Disagree; 2 = Disagree; 3 = Neutral; 4 = Agree; 5 = Strongly Agree

Statement	1	2	3	4	5
Humans are severely abusing the environment.	☐	☐	☐	☐	☐
The balance of nature is very delicate and easily disturbed.	☐	☐	☐	☐	☐
Humans have the right to modify the natural environment to meet their needs.*	☐	☐	☐	☐	☐
We are approaching the limit of the number of people the Earth can support.	☐	☐	☐	☐	☐
Plants and animals have as much right to exist as human beings.	☐	☐	☐	☐	☐
Despite our special abilities, humans are still subject to the laws of nature.	☐	☐	☐	☐	☐

** Reverse-scored item*

Section D: Inclusion of Nature in Self Scale (INS)

On a scale from 1 (completely separate) to 7 (completely overlapping), to what extent do you feel connected to nature?

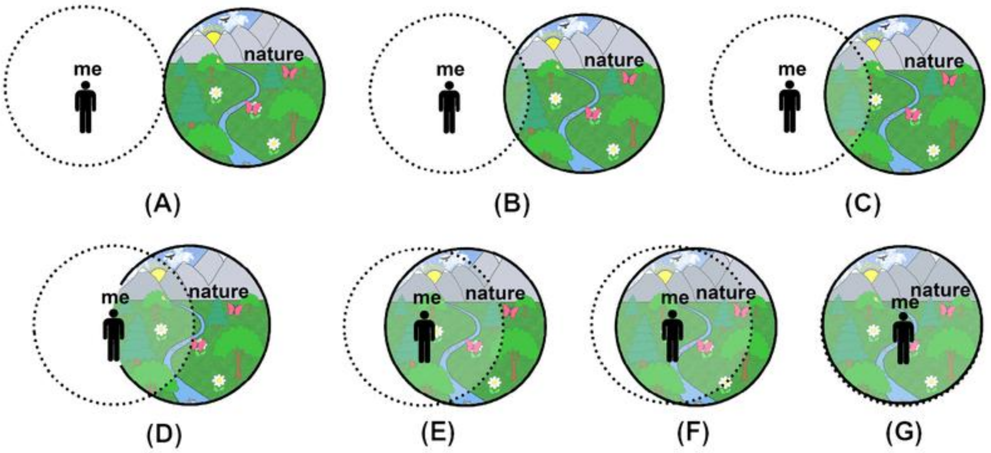

Circle your response: 1 - 2 - 3 - 4 - 5 - 6 - 7

Additional INS Dimensions

Scale: 1 = Not at all; 2 = A little; 3 = Moderately; 4 = Quite a bit; 5 = Very much

Statement	1	2	3	4	5
To what extent do you feel that your personal identity is connected to nature?	☐	☐	☐	☐	☐
How much time do you spend in natural environments outside of work?	☐	☐	☐	☐	☐

Section E: Organizational Alignment

Do you consider that [blinded for review] entertainment activities are compatible with conservation objectives?

- [ ] Strongly disagree
- [ ] Partially disagree
- [ ] Neither agree nor disagree
- [ ] Partially agree
- [ ] Strongly agree

Do you consider that [blinded for review] values and identity are somehow reflected in [Blinded for review]'s performance regarding Conservation?

- [ ] Strongly disagree
- [ ] Partially disagree
- [ ] Neither agree nor disagree
- [ ] Partially agree
- [ ] Strongly agree
